# Effects of elevated ultraviolet radiation on primary metabolites in selected alpine algae and cyanobacteria

**DOI:** 10.1016/j.jphotobiol.2015.05.016

**Published:** 2015-08

**Authors:** Anja Hartmann, Andreas Albert, Markus Ganzera

**Affiliations:** a Institute of Pharmacy, Pharmacognosy, University of Innsbruck, 6020 Innsbruck, Austria; b Research Unit Environmental Simulation, Institute of Biochemical Plant Pathology, Helmholtz Center Munich, 85764 Neuherberg, Germany

**Keywords:** Alpine algae, Cyanobacteria, Nucleosides, UV-exposure, Sun simulator, Primary metabolites

## Abstract

Extremophilic green algae and cyanobacteria are the most abundant species in high mountain habitats, where rough climate conditions such as temperature differences, limited water retention and high ultraviolet (UV) radiation are the cause for a restricted biological diversity in favor of a few specialized autotrophic microorganisms. In this study, we investigated four algal species from alpine habitat in a sun simulator for their defense strategies in response to UV-A radiation (315–400 nm) up to 13.4 W/m^2^ and UV-B radiation (280–315 nm) up to 2.8 W/m^2^. Besides changes in pigment composition we discovered that primary polar metabolites like aromatic amino acids, nucleic bases and nucleosides are increasingly produced when the organisms are exposed to elevated UV radiation. Respective compounds were isolated and identified, and in order to quantify them an HPLC-DAD method was developed and validated. Our results show that especially tyrosine and guanosine were found to be generally two to three times upregulated in the UV-B exposed samples compared to the non-treated control.

## Highlights

• Primary metabolites increased in algae and cyanobacteria after UV irradiation.
• Amino acids, nucleic bases and nucleosides were identified and quantified by HPLC.
• Tyrosine and guanosine were primarily upregulated (up to 3-fold).

## 1 Introduction

Sunlight at the earth’s surface, consisting of UV-B radiation (280–315 nm), UV-A radiation (315–400 nm), photosynthetically active radiation (PAR, 400–700 nm) and infrared radiation (>700 nm), is an important factor for the survival of high alpine algae, which are additionally exposed to harsh abiotic conditions such as temperature changes, extreme weather conditions and short vegetation periods [1]. As Blumthaler et al. have reported previously, the exposure to harmful irradiation increases dramatically with altitude under clear sky conditions [2]. Per 1000 m they measured 9% higher UV-A levels and an increased UV-B radiation by 18%.

Furthermore, environmental pollution primarily due to the use of chlorofluorocarbons has promoted depletion of the stratospheric ozone layer, which resulted in higher harmful short wave UV-B irradiation on the earth’s surface. Although these compounds were banned in the Montreal Protocol almost three decades ago, the problem persists possibly due to rising concentrations of greenhouse gasses [3]. Adaptation strategies are therefore required for any living organism and they have extensively been studied for higher plants [4], [5]. In algae and cyanobacteria several adaptation mechanisms to ultraviolet stress are known [6], [7], for example self-shading by mat formation [8], migration to higher water depth (lower UV-levels) [9], the development of anti-oxidative systems involving enzymatic and non-enzymatic strategies [10], or the synthesis of special secondary metabolites including mycosporine-like amino acids [11], [12], scytonemin [13], [14] and pigments [15], [16]. The latter are the best studied components and we also observed a rise in pigment levels after UV exposure. Yet, very little is known for algae and cyanobacteria from high mountain habitat, especially concerning alteration in primary metabolites. In our study we investigated the effects of elevated UV radiation on four respective species, *Pseudomuriella engadiensis*, *Coelastrella terrestris*, *Calothrix* sp. and *Leptolyngbya foveolarum* from high mountain habitat. The most significant changes were noticed concerning highly polar compounds, which showed to be nucleosides and aromatic amino acids. Till date only a few analytical studies described the determination of nucleosides in medicinal plants [17], [18]. However, to the best of our knowledge this study is the first report on the quantification of these metabolites in algae and cyanobacteria.

## 2 Materials and methods

### 2.1 Reagents and chemicals

All solvents used for isolation and analytical studies (methanol, acetonitrile, dichloromethane and glacial acetic acid) were of analytical grade and purchased from Merck (Darmstadt, Germany). Inorganic salts and vitamins for the culture media, as well as the nucleic bases uracil (1), thymine (3), adenine (6) and the nucleosides inosine (5), guanosine (7) and adenosine (9), and Sephadex-LH 20 material, used for isolation purposes, were purchased from Sigma Aldrich (Vienna, Austria). Standards of amino acids namely l-tyrosine (2), dl-phenylalanine (4) and dl-tryptophan (8) were obtained from Serva Electrophoresis (Heidelberg, Germany). Soil for preparing an extract required in the algal culture media was commercially available material (Floragard flower soil, Oldenburg, Germany). HPLC grade water was produced by a Satorius arium 611 UV water purification system (Göttingen, Germany).

### 2.2 Biological material and cultivation methods

Two green algae (*P. engadiensis* (former name: *Bracteacoccus engadiensis*) and *C. terrestris*) and two cyanobacteria (*Calothrix* sp., *L. foveolarum*) were investigated in this study. *P. engadiensis* (221-3; GenBank: KF673365.1) was purchased from the EPSAG Culture Collection of Algae (University of Göttingen, Germany) and *C. terrestris* (H4403; GenBank: JX513888.1) from the Culture Collection of Algae at the Charles University in Prague, Czech Republic. *Calothrix* sp. (034; GenBank: HM751858.1) and *L. foveolarum* (132; GenBank: AM398970.1) were ordered at the Culture Collection of Autotrophic Organisms in Třeboň, Czech Republic. Cultivation of the pure-culture strains was carried out in Erlenmeyer flasks, either using Bold’s Basal medium (for green algae) [19] or BG-11 media (for cyanobacteria) [20]. The flasks were kept under controlled and optimal growth conditions of 20 °C. PAR was provided from 18 W lamps (220–240 V; 50/60 Hz) following a light–dark rhythm of 16:8. In the exponential growth phase each culture was transferred into fresh media.

### 2.3 UV treatment

**Fig. 1:**
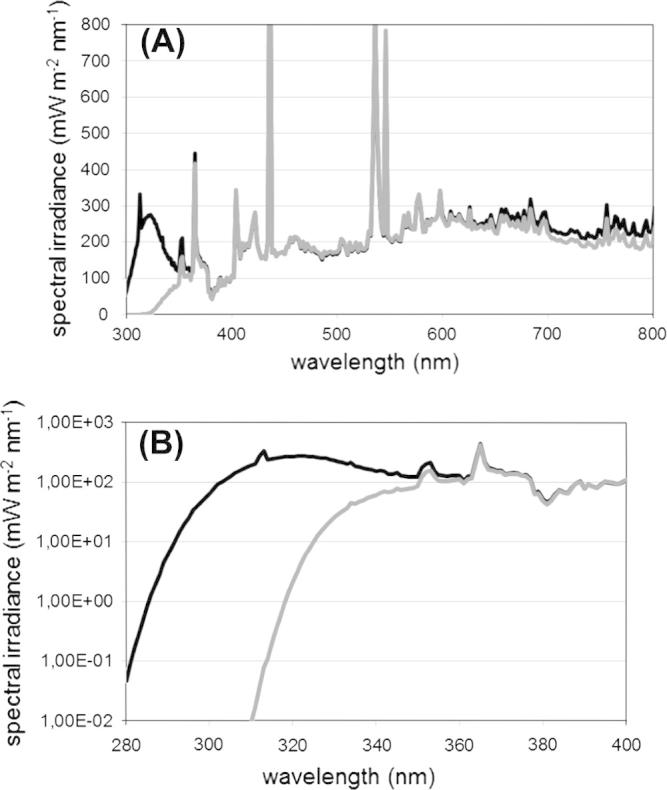
Sun simulator spectra for control (gray line) and treatment (black line) during the experiments; (A) spectra covering the range 300–800 nm; and (B) detail representing the relevant range from 280 to 400 nm.

The irradiation of the algae was carried out in the sun simulator of the Helmholtz Center in Munich, Germany. The simulated photobiological conditions provided exposure very close to global solar radiation from UV to near infrared radiation using a combination of four different types of lamps (metal halide lamps, quartz halogen lamps, blue fluorescence tubes, and UV-B fluorescence tubes). The arrangement of many of these lamps in several groups allowed us to simulate also the diurnal variation of solar radiation by switching on and off appropriate groups. Soda-lime and acrylic glass filters were used to prevent the biological material from damage by UV-C radiation (100–280 nm), emitted by UV-B fluorescence tubes, and to adjust the short-wave cut-off in the UV-B spectrum [21], [22], [23]. The UV exposure of the algae was performed in a sun simulator, which was divided into two compartments. The right part was covered by soda-lime and acrylic glass filters to allow exposure to UV-A, UV-B radiation and PAR, whereas the left part was covered by normal float glass to eliminate UV-B radiation. The experiments were carried out for 3 days using the first 24 h as an adaptation phase for the organisms. In this period no UV-B radiation was supplied (control). Irradiation experiments were then subsequently carried out on day two and three, always providing 14 h of UV-B irradiation per day. Additionally, a light–dark cycle of 16:8 h was set, i.e. PAR was switched on one hour before the onset of UV-B radiation and switched off one hour after UV-B exposure. During the UV-B treatment the algae were exposed to elevated UV-B radiation of 2.8 W/m^2^ and UV-A radiation of 13.4 W/m^2^, whereas the control only obtained UV-A radiation of 7.0 W/m^2^. Maximum PAR was set to 340 μmol/m^2^/s during UV-B exposure (Fig. 1). Temperature was controlled at 20 °C and a constant relative air humidity of 90% was adjusted to avoid desiccation stress. The algae were exposed to these conditions in open glass petri dishes (180 × 30 mm; Steriplan, VWR, Vienna, Austria). Prior to the experiments the algae culture broth was equally divided into 15 petri dishes for each species, during the experiments Bolds Basal culture medium was regularly added to keep the liquid level in each petri dish constantly at 1 cm. After 24 h (adaption phase) 3 petri dishes were removed and used as a non-irradiated control. Subsequently, after 48 h and 72 h again 3 petri dishes were removed from each compartment; the study design is shown in Fig. S1 (supplementary material).

### 2.4 Sample preparation

Samples, i.e. UV-irradiated material and non-irradiated control samples of all species, were directly filtered after removal from the chamber through pre-weighted glass fiber filters (Whatman GF/C; VWR) using a vacuum pump. For each petri dish 6 filters were used. The filters were freeze-dried (Heto power dry PL 6000, Thermo Fisher Scientific, Waltham, USA) for 48 h and their dry weight was determined. The dried filters were ground in a Micro-Dismembrator (Sartorious) in pre-cooled Teflon jars and extracted with methanol/water (25:75) in an ultrasonic bath (Bandelin Sonorex 35 kHz, Berlin, Germany) for 15 min at 45 °C. After centrifugation at 3000 rpm for 10 min, the supernatant was collected and evaporated at 45 °C in a rotary vacuum evaporator (Büchi, Flawil, Switzerland). For storage at −20 °C these extracts were transferred in screw-cap glasses and again lyophilized. For HPLC analysis solutions with a concentration of 10 mg/ml were prepared in water.

### 2.5 Initial screening by HPLC

For preliminary studies the irradiation experiments were applied only on *L. foveolarum* following the conditions described in Section 2.3. The experiments should indicate which compound class is primarily affected by the UV-treatment. Accordingly, after harvesting the material it was extracted with solvents ranging from low to high polarity; respective HPLC chromatograms are shown in Fig. S2 (supplementary material).

### 2.6 Isolation of guanosine, tyrosine and uracil

The 25% methanol extracts of irradiated *L. foveolarum* (2.8 W/m^2^ UV-B and 13.4 W/m^2^ UV-A, for 3 days) were merged, and fractionation was started on a Sephadex-LH-20 column using methanol as mobile phase, resulting in 10 fractions. They were assayed by HPLC and from fractions 8 and 9 uracil (1) and tyrosine (2) could be isolated by preparative HPLC. A description of the isolation protocol is provided as supporting information (Fig. S3). Following the same strategy guanosine (7) was isolated from non-irradiated and commercially available *Chlorella* sp. material (LOT-Nr. L310004, Naturprodukte Lembcke, Faulenrost, Germany); this species was not included in further irradiation experiments. The structure of all isolated substances was confirmed by LC–MS and NMR experiments, which were in good agreement to reported data [24], [25], [26]. NMR studies were performed on an Ultra-Shield 600 MHz instrument from Bruker (Rheinstetten, Germany), compounds were dissolved in deuterated water or methanol, and tetramethylsilane (all from Euriso-Top, St Aubin Cedex) was used as internal standard. NMR-shift values of the isolated compounds are summarized in the Table S1 (supplementary material).

### 2.7 Analytical conditions

Experiments were performed on an Agilent 1200 HPLC system (Waldbronn, Germany), using a Triart C-18 column (150 × 3.00 mm, 3 μm) from YMC (Dinslaken, Germany). The mobile phase consisted of 10 mM ammonium acetate in water (A) and 10 mM ammonium acetate in acetonitrile (B). The components were eluted by using the following solvent gradient: 0–16 min 2% B, 16–25 min 2–16% B, 25–35 min 16–98% B. Then the column was re-equilibrated for 20 min with 2% B. The DAD was set to 260 nm except for tyrosine (2) and phenylalanine (4), which both were monitored at 210 nm. Flow rate, injected sample volume and column temperature were adjusted to 0.3 ml/min, 10 μl and 20 °C, respectively.

### 2.8 Method validation

**Fig. 2:**
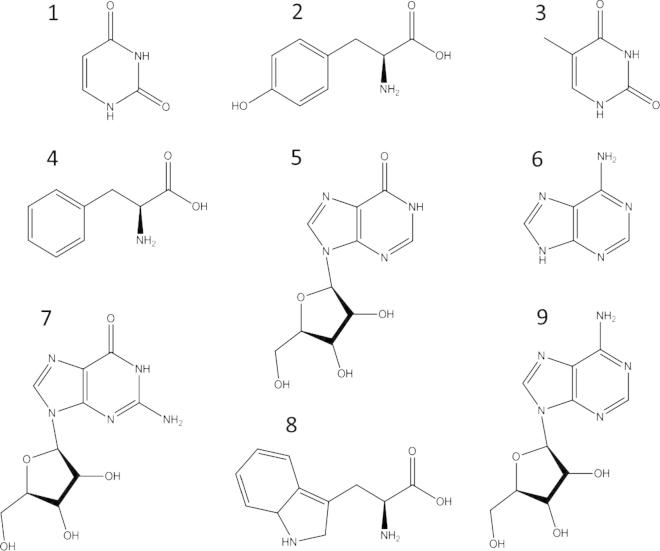
Structures of the investigated analytes, numbered in their HPLC elution order.

For all nine standard compounds (see Fig. 2 for structures) calibration curves were established. By accurately weighing and dissolving them in water a single stock solution with a concentration of 100 μg/ml each was obtained. This solution was stable at least for 7 days if stored at 4 °C. Eleven calibration levels were prepared from the stock solution, and each of them analyzed under optimum HPLC conditions in triplicate. Limit of detection (LOD) and limit of quantification (LOQ) were visually evaluated corresponding to concentrations equivalent to S/N ratios of 3 (LOD) and 10 (LOQ). Accuracy was determined by spiking non-irradiated *L. foveolarum* samples with known amounts of the reference compounds at three concentration levels. The methods precision was confirmed by repeated analysis of a sample solution on one day (inter-day precision) and by its re-analysis on three consecutive days (intra-day precision).

## 3 Results

### 3.1 Identification of compounds

As it was not known which metabolites change to the highest degree under UV-stress, extracts of irradiated *L. foveolarum* material were prepared using different solvents, containing mainly polar (25% methanol), medium polar (methanol) or non-polar constituents (DCM). The respective HPLC-chromatograms were compared to those of a control sample exposed to UV-A radiation only. The results indicated changes in polar as well as non-polar metabolites, whereby the latter was expected as carotenoids and other pigments are known photo protectants. Their tentative assignment in the dichloromethane extracts was possible based on typical UV spectra and LC–MS data (Fig. S4 in supplementary material). More surprising were drastic changes in the composition of highly polar compounds (Fig. S2C), which isolation was challenging because the available starting material was limited, and standard procedures like silica gel chromatography could not be utilized. Yet, it was possible to isolate and elucidate the chemical structure of three of them; they showed to be guanosine, uracil and tyrosine. This was an unexpected observation, because in algae the increased synthesis of primary metabolites in response to UV radiation has not been reported till date. As a consequence, to study these effects in detail other algae from the same habitat (*P. engadiensis*, *C. terrestris* and *Calothrix* sp.) were selected and exposed to sun-simulation experiments under controlled conditions.

### 3.2 Development and validation of the HPLC method

**Fig. 3:**
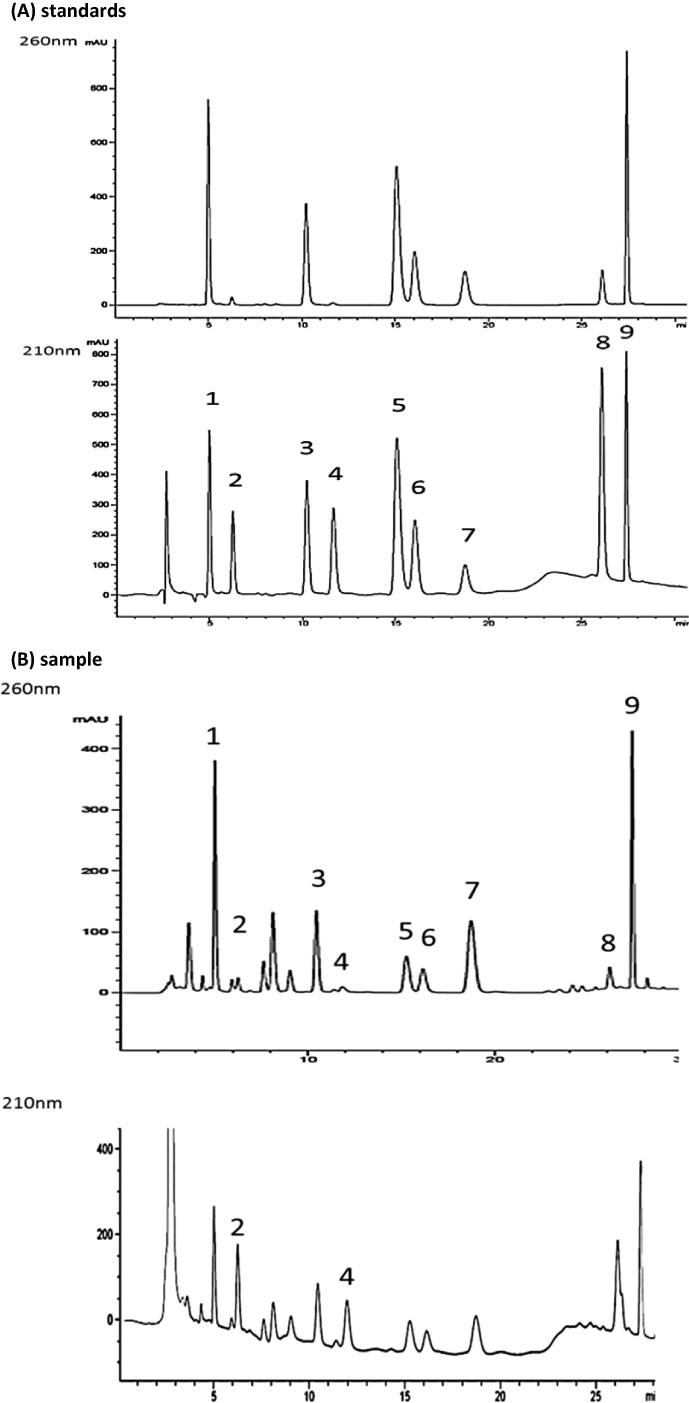
HPLC separation of the nine standard compounds (A) and their assignment in one sample (25% methanolic extract of *C. terrestris*, irradiated for 1 day with UV-B: 2.8 W/m^2^ and UV-A: 13.4 W/m^2^; (B)); peak assignment: uracil (1), l-tyrosine (2), thymine (3), dl-phenylalanine (4), inosine (5), adenine (6), guanosine (7), dl-tryptophan (8) and adenosine (9).

To permit meaningful conclusions the number of investigated compounds was increased to nine (aromatic amino acids, nucleic bases and nucleosides). Their polar nature renders an analysis by reversed phase chromatography difficult, and most screened stationary phases resulted in poor retention or partial overlap of the compounds of interest. From the tested columns only one (Triart C-18) showed satisfactory results, but only after all relevant separation parameters were optimized. The use of acetonitrile and a buffer (10 mM ammonium acetate with pH 4.75) were required for acceptable peak shape, and a highly aqueous gradient for retention. The latter was further improved by setting the column temperature to 20 °C. This setup permitted the separation of all 9 standards in less than 30 min (Fig. 3).

As quantitative studies were attempted the developed HPLC assay had to be validated, whereby the respective ICH guidelines were followed. The validation results are summarized in Table 1. For all analytes a linear range from at least 94.9 to 4.92 μg/ml was obtained, with excellent correlation coefficients between 0.999 and 1.000. The limits of detection and limits of quantification were found to be from 0.08 to 1.43 μg/ml and from 0.23 to 4.35 μg/ml, respectively. Selectivity of the method was assured by no visible co-elutions (shoulders) in the relevant signals, and by very consistent UV spectra within the respective peaks. The latter was confirmed by using the peak-purity option in the operating software. Intra-day (RSD: 0.20–4.03%) and inter-day precision (RSD: 0.45–4.03%) were well acceptable too, only inosine showed a higher intra-day variation of 9.21%. Accuracy was assured by spiking accurately weighted samples of *L. foveolarum* with three different concentrations of the standards. For nearly all compounds the observed recovery rates were between 94.1% and 100.8%. Inosine was again an exception, which possibly is due to its unstable nature and decomposition during extraction. This effect however, has been described in literature for other matrices already [27].

**Table 1:** Summary of validation parameters.

Substance	Regression equation	Correlation coefficient	Range (μg/ml)	LOD (μg/ml)	LOQ (μg/ml)	Precision (intra-day) RSD%	Precision (inter-day) RSD%	Accuracy (high spike)	Accuracy (medium spike)	Accuracy (low spike)
Uracil	*y* = 125.70*x* + 45.86	*R*^2^ = 0.9999	103.7–2.59	0.21	0.65	0.68	1.50	98.22	99.63	98.33
Thymine	*y* = 107.72*x* + 0.37	*R*^2^ = 1.0000	105.1–1.31	0.09	0.28	1.18	1.42	98.66	98.05	95.46
Adenosine	*y* = 98.48*x* + 12.52	*R*^2^ = 1.0000	100.3–1.25	0.08	0.23	0.20	4.03	99.41	98.89	101.21
Guanosine	*y* = 75.15*x* − 7.03	*R*^2^ = 1.0000	96.3–1.20	0.14	0.44	1.16	2.98	94.08	98.64	97.23
Adenine	*y* = 57.03–9.21	*R*^2^ = 0.9999	97.6–2.44	0.42	1.26	1.06	2.25	98.59	99.83	96.62
Inosine	*y* = 167.95–10.69	*R*^2^ = 1.0000	98.5–1.23	0.08	0.25	0.45	9.21	82.58	82.59	78.46
Tryptophan	*y* = 34.68*x* − 0.76	*R*^2^ = 1.0000	94.9–1.19	0.13	0.39	1.05	2.66	100.15	100.66	98.85
Phenylalanine	*y* = 59.54*x* + 31.09	*R*^2^ = 0.9995	98.4–4.92	1.43	4.35	2.02	0.45	100.77	99.22	98.93
Tyrosine	*y* = 5.91*x* − 1.26	*R*^2^ = 0.9996	95.1–1.19	0.99	2.84	4.34	2.93	99.97	99.89	99.70

### 3.3 Sun simulation experiments

**Fig. 4:**
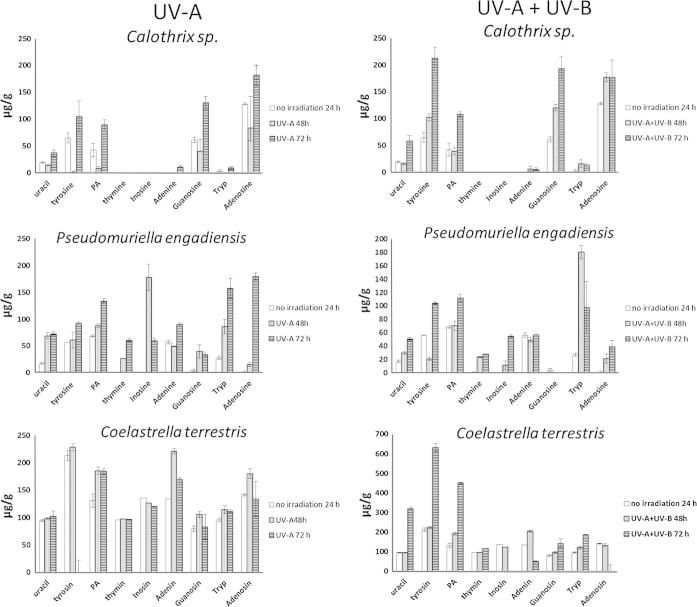
Quantitative determination of selected amino acids, nucleic bases and nucleosides in samples of *Pseudomuriella engadiensis*, *Coelastrella terrestris*, *Calothrix* sp. before and after exposure to UV.

The amount of each selected nucleic base, nucleoside and aromatic amino acid in *P. engadiensis*, *C. terrestris* and *Calothrix* sp. was determined by HPLC; *L. foveolarum* could not be studied in detail as all respective material was used for isolation. Each sample was exposed to elevated levels of UV-A radiation alone or in combination with UV-B radiation, exposure time was one or two days. For each experiment 3 samples per algae were available, they were individually extracted and analyzed by HPLC (sample concentration 10 mg of extract in 1 ml water). As Fig. 4 and Table S2 show, each alga responded differently to the exposure. However, certain trends were comparable in all of the three species. For example, generally higher amounts of uracil, tyrosine, phenylalanine and tryptophan were found after treatment with UV-A and UV-B radiation at 72 h. Neither in the irradiated nor in the control samples of the investigated cyanobacterium (*Calothrix* sp.) detectable amounts of thymine and inosine were found. Tryptophan (2.15 μg/g in the control sample) and adenine (up to 9.89 μg/g) were present only in rather small amounts. However, guanosine and adenosine were found to be upregulated after both irradiation experiments, thus UV-A as well as UV-B radiation seemed to increase their synthesis in *Calothrix* sp. For example, the amount of guanosine in the non-irradiated samples was determined to be 61.3 μg/g, while after two days of UV-A treatment it increased to 130.2 μg/g, showing an even higher content after two days of combined UV-A and UV-B exposure (193.2 μg/g). This is equivalent to a more than three-fold increase compared to the control. With regard to the aromatic amino acids investigated, also the amount of tyrosine and phenylalanine was highly upregulated in all species, especially in those experiments which supplied UV-B radiation as well. For instance, the concentration of tyrosine increased several times in the treated samples (72 h exposure to UV-A and UV-B radiation: *Calothrix* sp.: 212.8 μg/g, *C. terrestris*: 632.0 μg/g) compared to the non-irradiated material (*Calothrix* sp.: 64.6 μg/g, *C. terrestris*: 213.0 μg/g). The concentrations of thymine and adenine remained generally constant and did not seem to be significantly influenced by UV treatment. Overall, a 72 h UV exposure resulted in most significant changes, which were observed when the samples were treated with UV-A and UV-B radiation (*Calothrix* sp., *C. terrestris*) or UV-A radiation alone (*P. engadiensis*). The highest content of individual compounds (tyrosine) and in total was found in the green alga *C. terrestris*.

## 4 Discussion

Producing secondary metabolites like mycosporine like amino acids or pigments in response to UV radiation is a well-known protection mechanism in algae and cyanobacteria [11], [12], [13], [14], [15], [16]. Possible effects on primary metabolites such as carbohydrates, amino acids and nucleosides have not been reported to our knowledge, even though they are essential substrates for secondary metabolic pathways [28]. Results of our study support this statement, because at least some of the compounds which were found to be upregulated are precursors of known photoprotectants. For example, tyrosine and phenylalanine are required for melanin and bacterial pyomelanin biosynthesis [29], as well as for the formation of mycosporine like amino acids. Therefore, our data at least partially explains already known strategies and contributes to a holistic understanding of defense mechanisms of algae and cyanobacteria against UV stress.

A few studies could show an upregulation of primary metabolites in higher plants when they are exposed to UV radiation [28], [30]. However, as no such effects have been described for algae and cyanobacteria, our study is the overall first evidence for a similar strategy in these organisms. This is not only of relevance for developmental biology but also might pave way for new applications. Several studies have reported on antioxidative, DNA protective and anti-inflammatory effects of nucleosides; especially adenosine and guanosine seem to be beneficial in this respect [31], [32], [33]. Guanosine is able to prevent deamination of cytosine in DNA solutions and resulted in a significant decrease in hydrogen peroxide and hydroxyl radicals in mice [32]. If these cell protecting effects are relevant for other organisms, including the studied ones needs to be investigated. Adenosine depletion is also the consequence of tissue injury and inflammation [34]. Interestingly, in our study especially these two molecules showed to be strongly influenced by UV radiation, e.g. the adenosine content in *P. engadiensis* multiplied nearly 200-fold after UV-A exposure. An explanation for these observations might be effects on the formation of cAMP, a compound that is sensitive to UV-A and metabolized via 2′,3′-cAMP adenosine pathway to adenosine [35]. The cAMP formation has been confirmed in higher plants and cyanobacteria but not in algae [36], so that it remains speculative if the investigated species react in a similar way.

The possible use of these molecules or respective algal extracts for medicinal and cosmetic purposes is certainly worth a deeper investigation, especially as the mechanism by which the compounds modulate the immune system is still not clear [37]. Thus, further research on this topic may not only clarify possibly new protection strategies against UV irradiation but also contribute to health benefits in inflammatory diseases.

## Conflict of interest

The authors declare no conflict of interest.
