# Supplementary material for: Effects of elevated ultraviolet radiation on primary metabolites in selected alpine algae and cyanobacteria
Source: J Photochem Photobiol B. 2015 Aug;149:149–55. doi: 10.1016/j.jphotobiol.2015.05.016 (PMC4509709; doi:10.1016/j.jphotobiol.2015.05.016)

Effects of elevated ultraviolet radiation on primary metabolites in selected alpine algae and cyanobacteria

**SUPPLEMENTARY MATERIAL**

**Anja Hartmann^1^, Andreas Albert^2^**, **Markus Ganzera^1,*^**

**Affiliation**

^1^ Institute of Pharmacy, Pharmacognosy, University of Innsbruck, 6020 Innsbruck, Austria

^2^ Research Unit Environmental Simulation, Institute of Biochemical Plant Pathology, Helmholtz Center Munich, 85764 Neuherberg, Germany

**Fig. S1:**

*Workflow of irradiation experiments.*


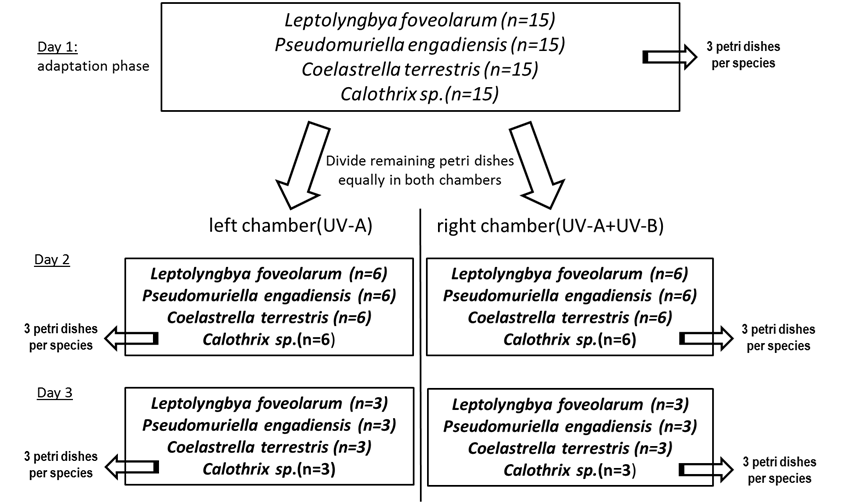


**Initial screening by HPLC**

Initially, the irradiation experiments were applied on *Leptolyngbya foveolarum,* following the conditions described in section 2.3. Each sample was consecutively extracted with three different solvents, 25 % methanol, methanol and dichloromethane, to obtain extracts of different polarity (Fig. S2). The extracts were evaporated and then analyzed by HPLC using a La Chrom Elite system (Hitachi, Tokyo, Japan). An YMC-Triat C18, (150 x 3.00 mm; 3 µm) column from was used for separation. The mobile phase comprised 0.1 % acetic acid in acetonitrile (A), methanol with 0.1 % acetic acid (B) and water with 0.1% acetic acid (C), applied in different gradients (table S3), depending on the type of extract. Detection was carried out at variable wavelengths from 200-700 nm, the injected sample volume was 10 µl, always with a sample concentration of 5 mg/ml.

**Fig S2:**

**A**) Changes in the methanolic extract *of Leptolyngbya foveolarum* after one and two days exposure to only UV-A radiation, or UV-A and UV-B radiation.

**
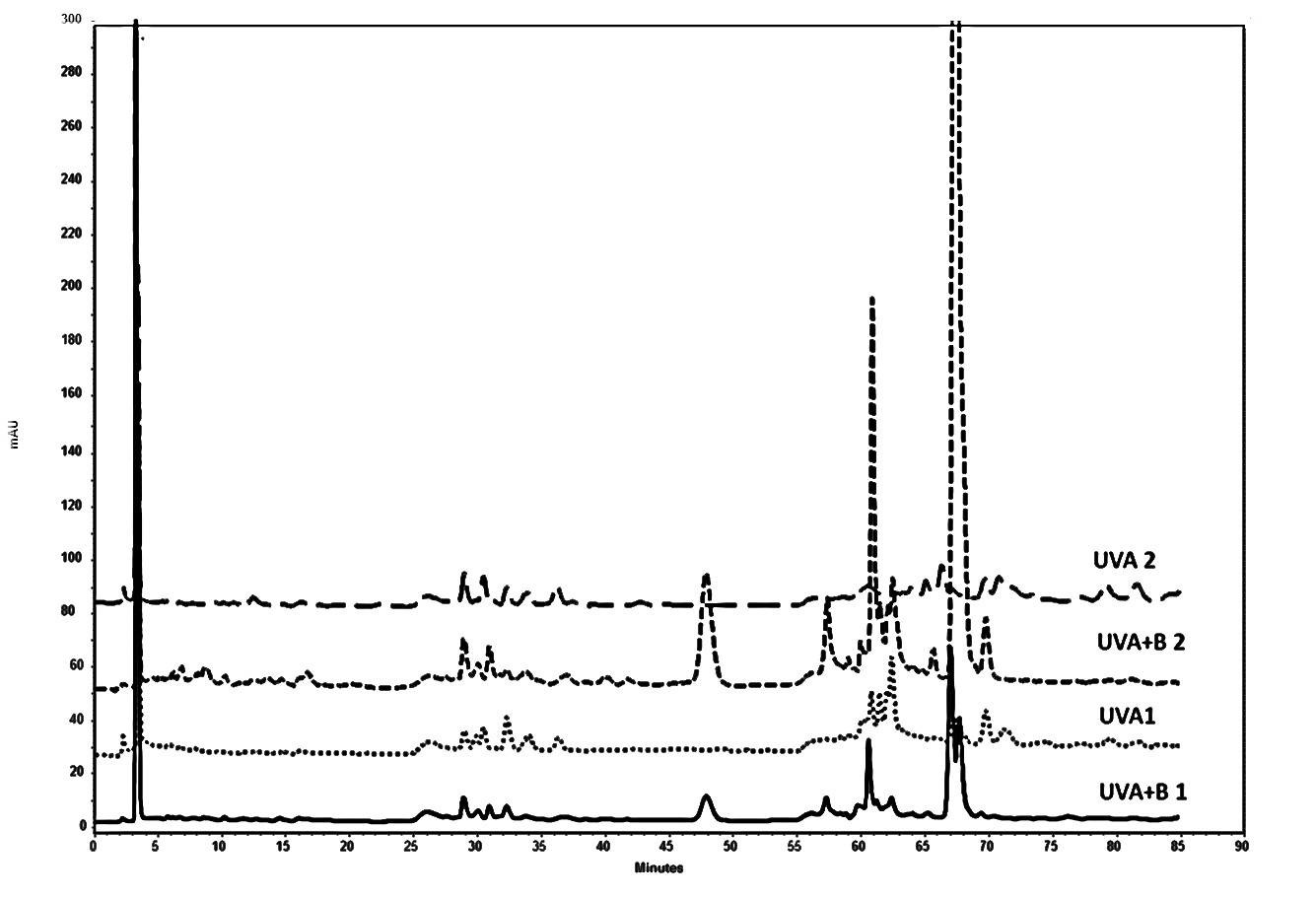
**

**B**) Changes in the dichloromethane extract *of Leptolyngbya foveolarum*. Comparison of UV-A vs UV-A + UV-B irradiated material (dashed line); 24 hours treatment. Major changes are indicated by arrows.


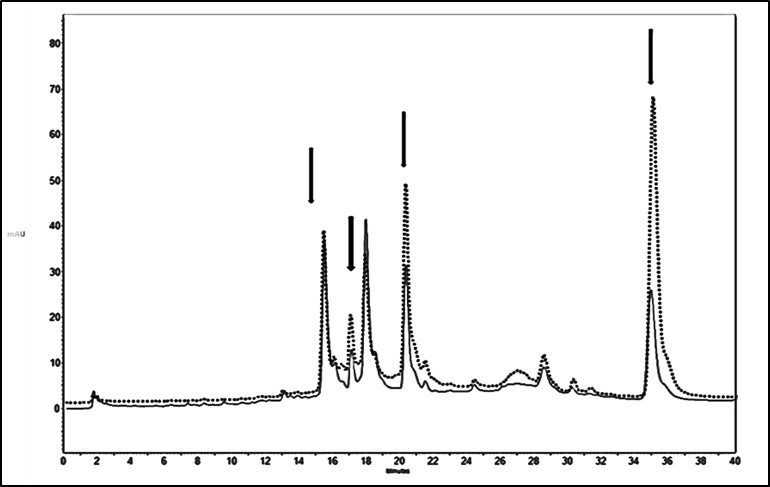


**C**) Changes in the 25 % methanolic extract of *Leptolyngbya foveolarum* (upper chromatogram shows the UV-A + UV-B irradiated sample, the lower one the control sample); 24 hours of treatment.


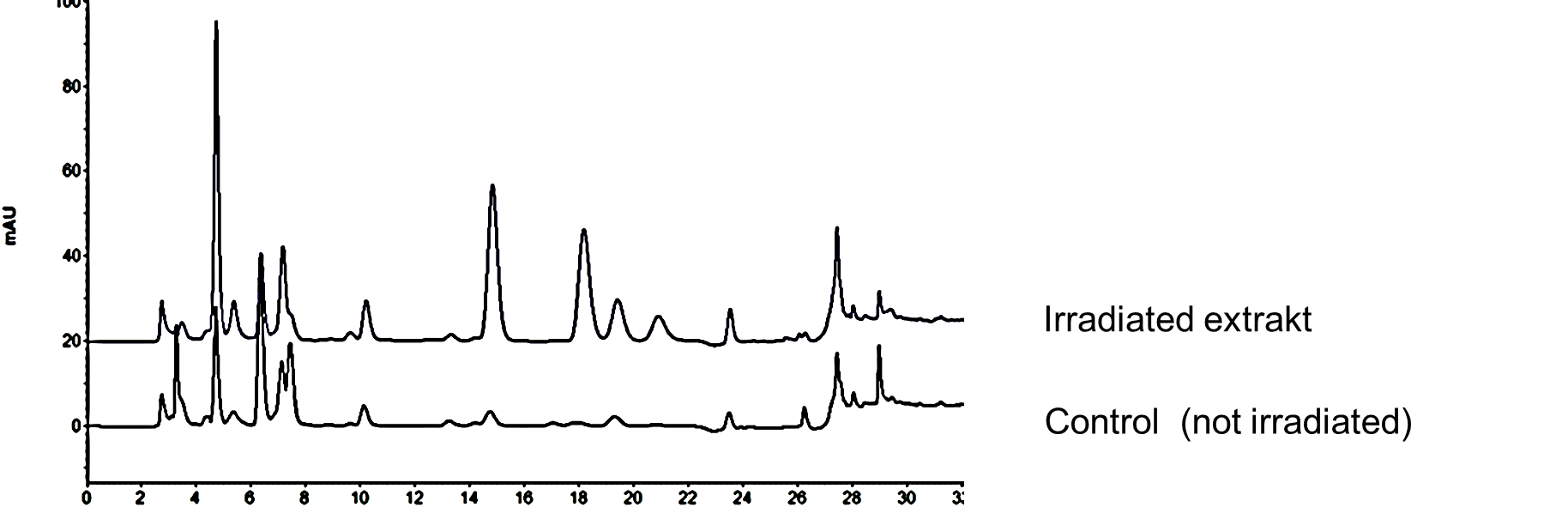


**Fig. S3:**

Isolation scheme for uracil (fraction 8) and tyrosine (fraction 9) from *L. foveolarum*. Isolated compounds are marked with arrow.


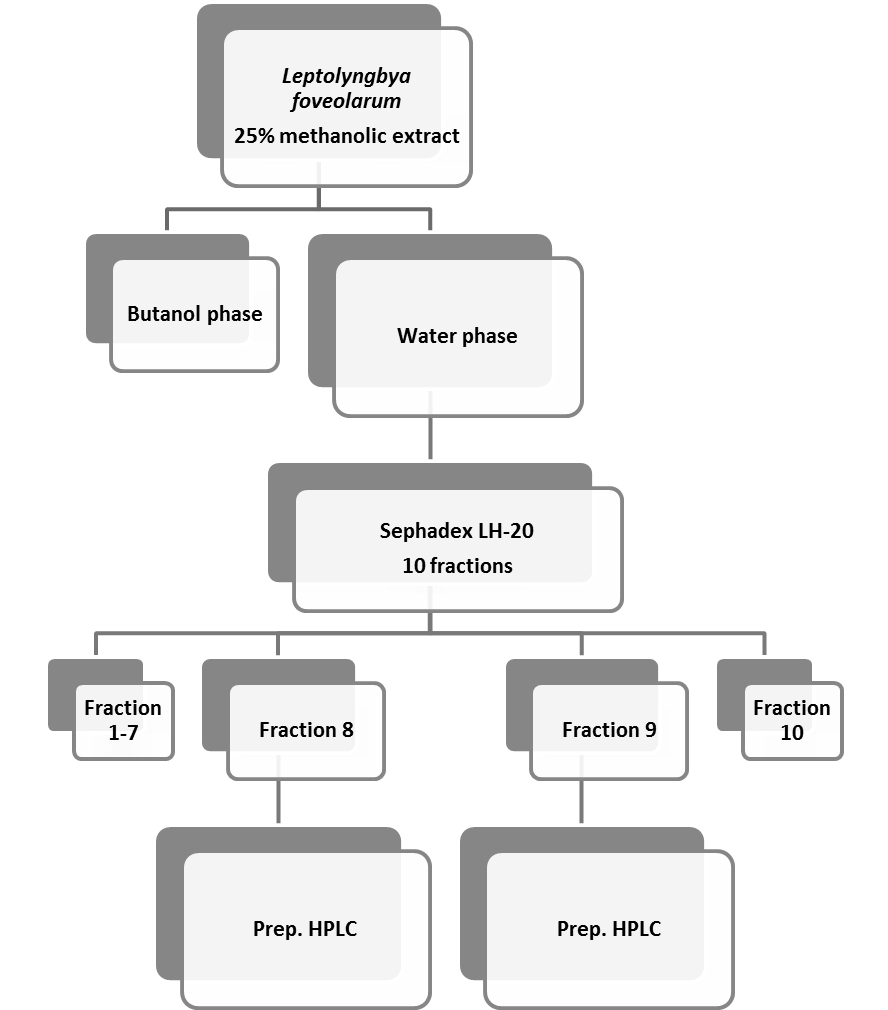


Fraction 8: Fraction 9:


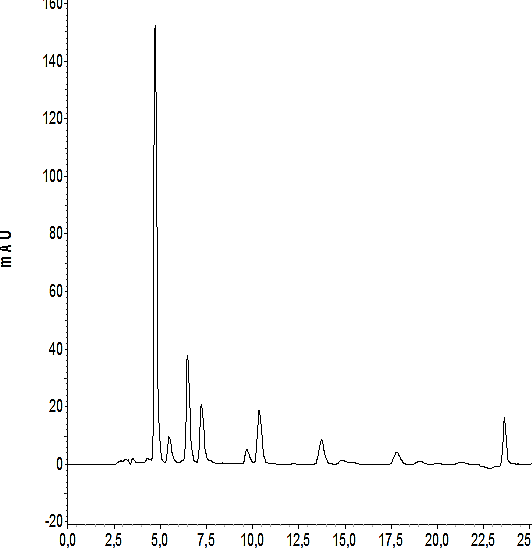

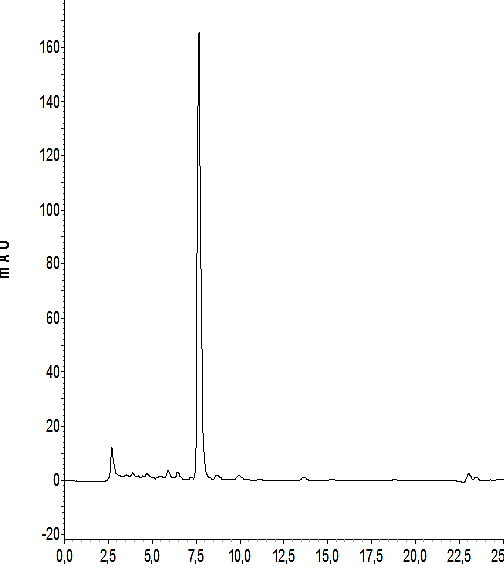


**Fig. S4**:

Pigment changes in algae due to UV-irradiation, as monitored in DCM extracts. 1 = cryptoxanthin, 2 = zeaxanthin, 3 = antheraxanthin, 4 = unidentified, 5 = lutein, 6,7 = α+β carotene. Peaks were tentatively assigned by LC-MS (fig. S3D) and UV-spectra.

**A)** *Pseudomuriella engadiensis*

**
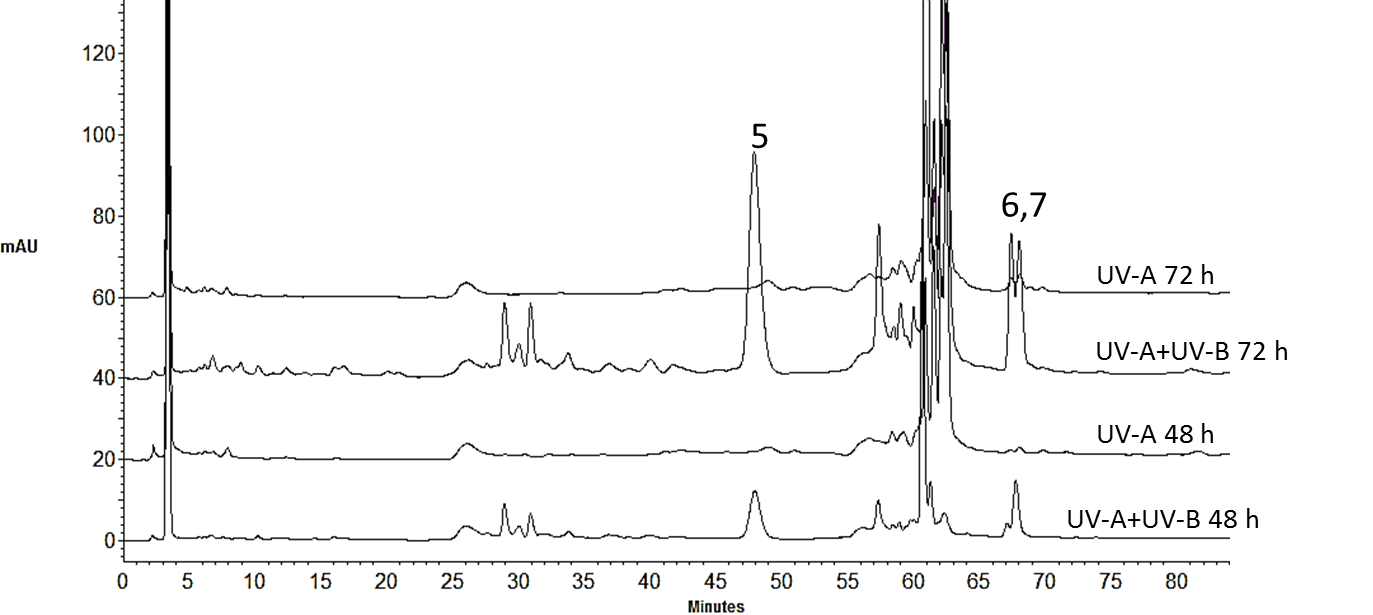
**

**B)** *Calothrix sp.*

**
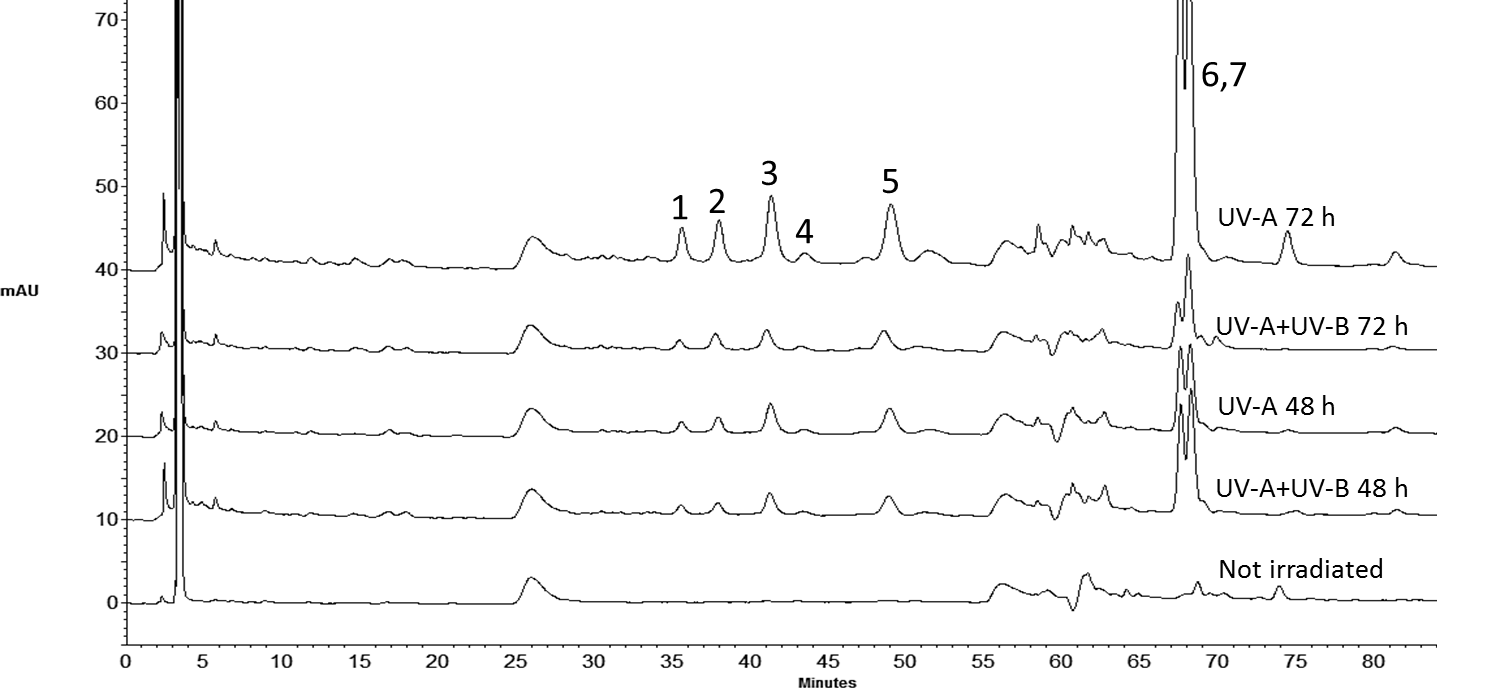
**

**C)** *Coelastrella terrestris* (same order of traces as in S3B)

**
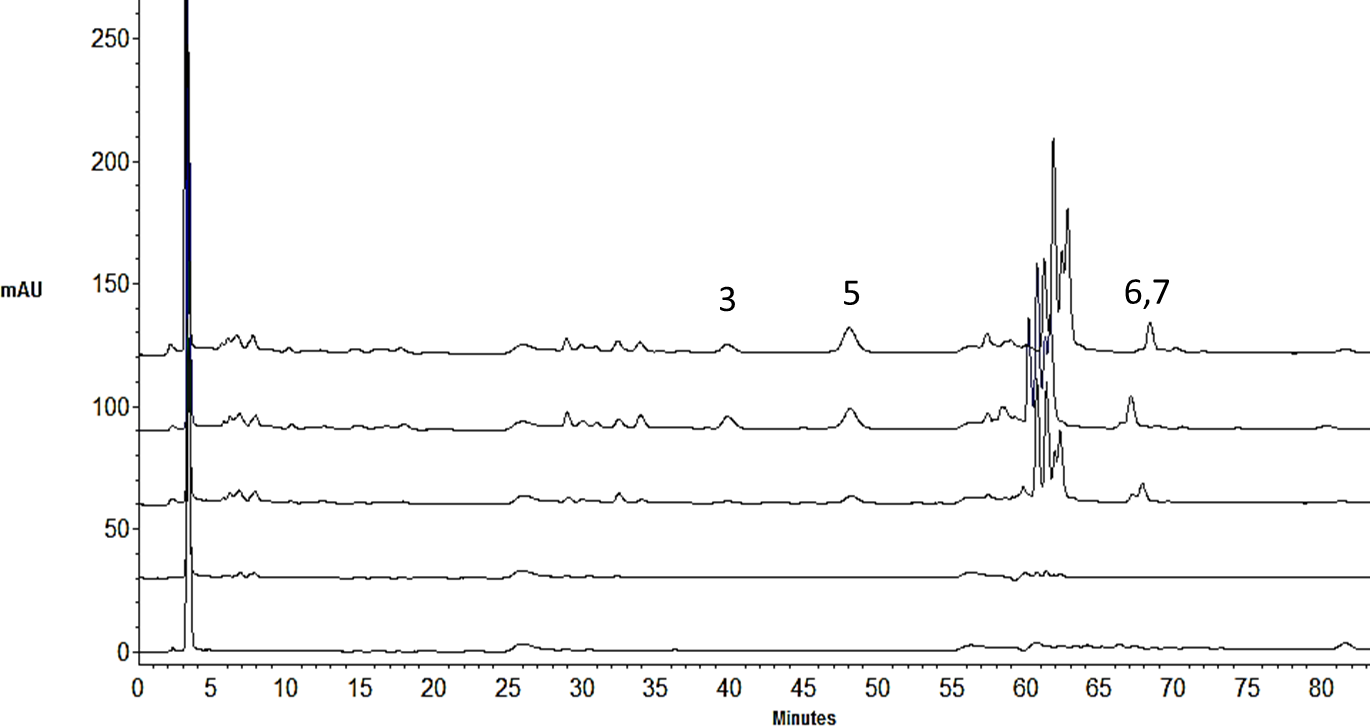
**

**D)** LC-MS data for peak assignment in *Calothrix sp.*


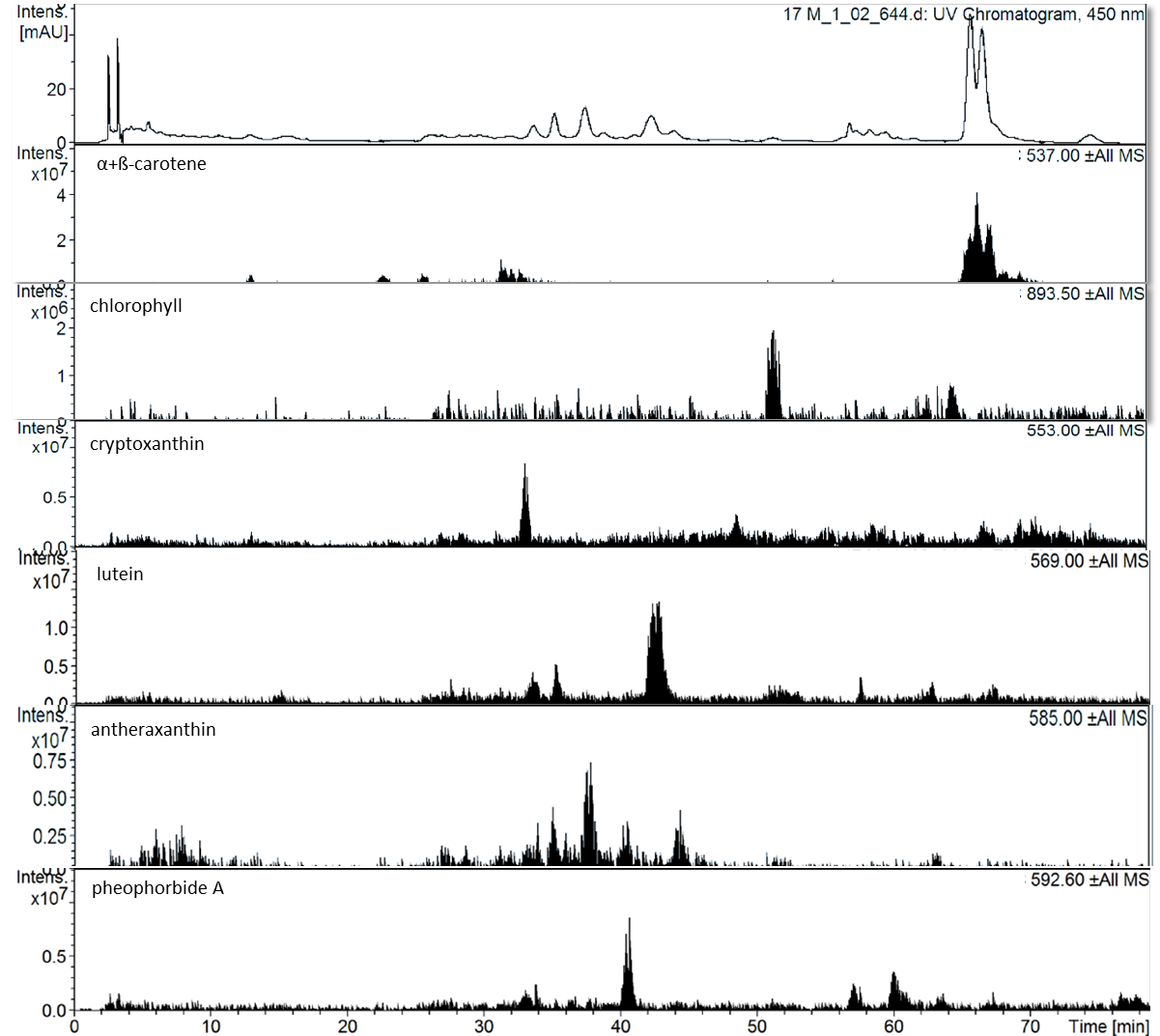


**Table S1:**

NMR-shift values of the isolated substances.


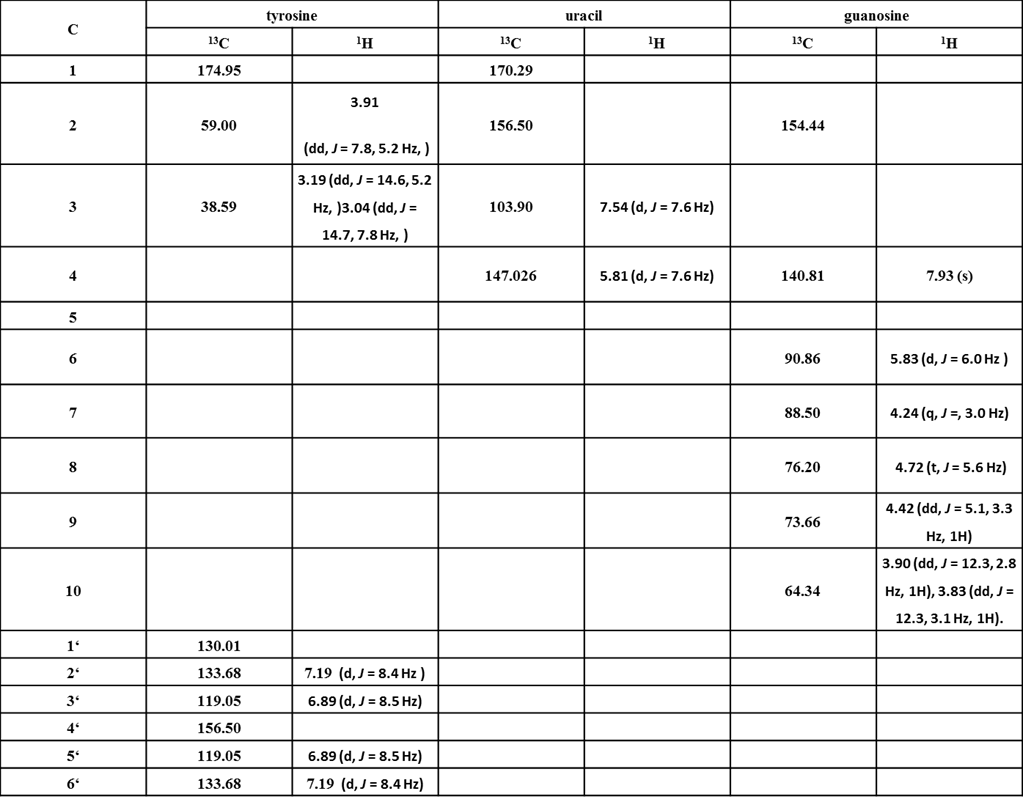


| tyrosine  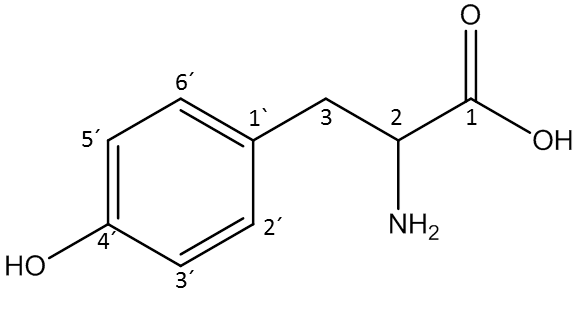 | uracil  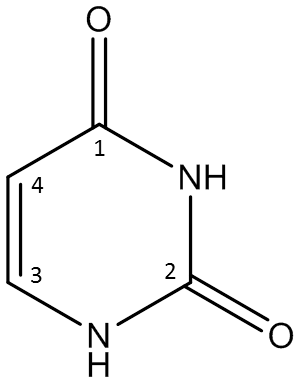 | guanosine  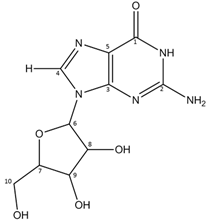 |
| --- | --- | --- |

The spectra of tyrosine and uracil were recorded in deuterated water, using TMS (tetramethylsilane) as internal standard, the spectra of guanosine was recorded in deuterated methanol. All samples were measured on an 600 MHz NMR instrument.

**Table S2:**

Quantitative results for radiated and non-radiated *Calothrix sp.* (**A**), *Coelastrella terrestris* (**B**) and *Pseudomuriella engadiensis* (**C**) samples (n=3).

**A)**

*
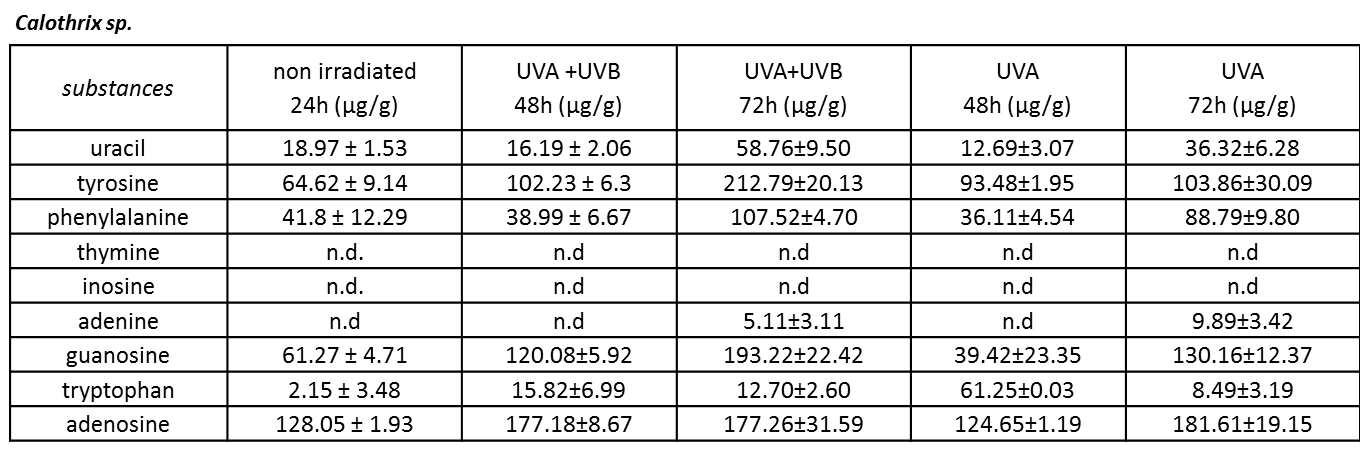
*

**B)**


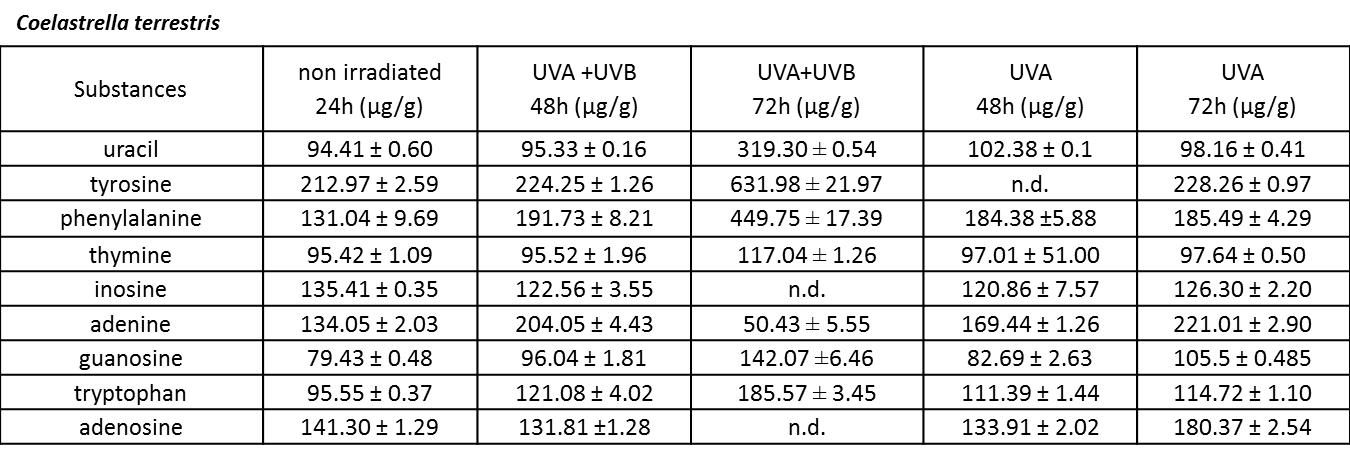


**C)**


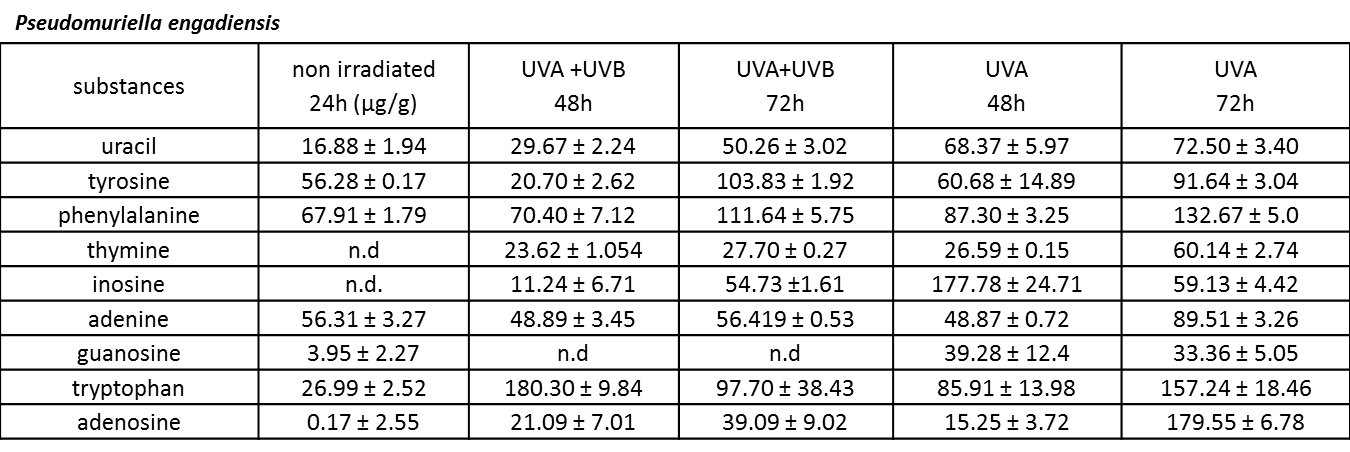


**Table S3:**

HPLC conditions for the analysis of different extracts (25 % methanol, methanol and dichloromethane) of irradiated *Leptolyngbya foveolarum* samples.


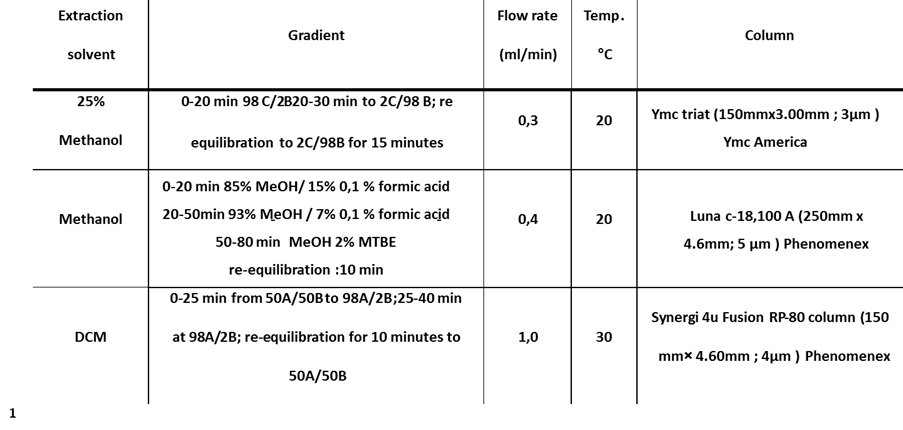

Supplement: Supplementary Figs. S1–S4 and Tables S1–S3 [file mmc1.docx]
